# CD7-positive leukemic blasts with *DNMT3A* mutations predict poor prognosis in patients with acute myeloid leukemia

**DOI:** 10.3389/fonc.2024.1342998

**Published:** 2024-03-21

**Authors:** Yanliang Bai, Xiaobai Sun, Mengyi Li, Xiaona Niu, Weijie Cao, Junwei Niu, Xingjun Xiao, Yuqing Chen, Kai Sun

**Affiliations:** ^1^ Department of Hematology, Zhengzhou University People’s Hospital and Henan Provincial People’s Hospital, Zhengzhou, China; ^2^ Department of Hematology, Nanyang Second General Hospital, Nanyang, China; ^3^ Department of Hematology, The First Affiliated Hospital of Zhengzhou University, Zhengzhou, China; ^4^ Department of Hematology, Beijing JiShuiTan Hospital, Capital Medical University, Beijing, China

**Keywords:** acute myeloid leukemia, CD7, Dnmt3a, immunophenotype, molecular diagnostics, prognosis

## Abstract

**Background:**

*DNMT3A* mutations can be detected in premalignant hematopoietic stem cells and are primarily associated with clonal hematopoiesis of indeterminate potential; however, current evidence does not support assigning them to a distinct European Leukemia Net (ELN) prognostic risk stratification. CD7 is a lymphoid antigen expressed on blasts in approximately 30% of acute myeloid leukemia (AML), and its role in AML remains unclear and depends on subgroup evaluation. This study investigated the prognostic value of *DNMT3A* mutation combined with CD7 expression in AML.

**Methods:**

We retrospectively analyzed the clinical data of 297 newly diagnosed non-M3 AML patients. According to the *DNMT3A* mutation and CD7 expression in AML cells, patients were divided into the *DNMT3A*-mutated/CD7-positive (CD7+), *DNMT3A*-mutated/CD7-negative (CD7-), *DNMT3A*-wild-type/CD7+, and *DNMT3A*-wild-type/CD7- groups.

**Results:**

The *DNMT3A*-mutated/CD7+ group had lower complete remission rates and higher relapse rates. Importantly, these patients had significantly shorter overall survival (OS) and relapse-free survival (RFS). Furthermore, multivariate analysis showed that CD7+ with *DNMT3A* mutation was an independent risk factor for OS and RFS.

**Conclusion:**

CD7+ with *DNMT3A* mutation predicts a poor prognosis in AML patients, and the immunophenotype combined with molecular genetic markers can help to further refine the current risk stratification of AML.

## 1 Introduction

Acute myeloid leukemia (AML) is a hematologic malignancy caused by abnormal clonal proliferation of hematopoietic stem and progenitor cells, and its clinical manifestations and prognosis are highly heterogeneous (1). Currently, the prognosis of AML patients is based mainly on cytogenetic and molecular genetic alterations (2). Although the relationship between some genetic mutations and the prognosis of AML has been clarified, the relationship between other genetic alterations and AML prognosis is not yet fully understood (3). *DNA methyltransferase 3A* (*DNMT3A*) is an epigenetic protease that, along with *DNMT3B*, is responsible for *de novo* DNA methylation (4, 5). At present, there is still some controversy about the prognostic significance of *DNMT3A* mutations. Most investigators contend that *DNMT3A* mutations are related to the poor prognosis of AML patients (6, 7). Moreover, some studies suggest that the poor prognostic value of *DNMT3A* mutations may be limited to specific types of AML patients (5, 8–11). However, the 2022 European Leukemia Net (ELN) guidelines state that current evidence is insufficient to classify *DNMT3A* mutations into a clear prognostic group (3). Therefore, more prognostic markers must be combined as a supplement to assess the prognostic value of *DNMT3A* mutations.

CD7 is a 40 kDa glycoprotein belonging to the immunoglobulin superfamily (12). CD7 is mainly expressed on the surface of T cells but can also be aberrantly expressed on AML blasts (13–15). In AML patients, the positive expression rate of CD7 is approximately 30% (16–18), but the prognostic significance of its expression has been inconsistently reported (17, 19–23), and further analysis and subgroup evaluation are needed.

The prognostic significance of *DNMT3A* mutation and CD7 expression in AML is still inconclusive, and there are currently no studies exploring the clinical significance of *DNMT3A* mutation combined with CD7 expression in AML patients. Therefore, we conducted a retrospective study to evaluate the prognostic value of *DNMT3A* mutation combined with CD7 expression in AML patients.

## 2 Patients and methods

### 2.1 Patients

In this retrospective analysis, 297 patients with *de novo* AML were enrolled. Samples were collected at Zhengzhou University People’s Hospital and the First Affiliated Hospital of Zhengzhou University from January 2017 to July 2021. The last date of follow-up was July 30, 2022. Patients with a history of hematologic disease, therapy-related AML or other malignancies, or acute promyelocytic leukemia (FAB-M3) were excluded. All baseline data were obtained from medical records, including age, sex, white blood cell count (WBC), hemoglobin (HB), platelet (PLT) count, lactate dehydrogenase (LDH), peripheral blood (PB) blast percentage, bone marrow (BM) blast percentage, immunophenotype, karyotype, gene mutations, and French-American-British classification (FAB) subtypes as well as the induction therapy regimen and treatment response. All patients signed an informed con-sent form at the time of treatment, and this study complied with the requirements of our institutional ethics committee (2021-140-02).

### 2.2 Immunophenotypic analysis

Before initial treatment, 2-3 ml of BM aspiration fluid was collected and placed in an EDTA anticoagulant tube. The Navios flow cytometer from Beckman Coulter Company in the United States was used for a 4-color immunolabeling method to detect the expression levels of CD2, CD4, CD7, CD34, CD56, CD38, CD117, CD33, CD13, HLA-DR, and others. CD45/SSC was utilized to set the gate, and the Kaluza software was employed to perform immunophenotypic analysis of BM blasts. Antigen expression was defined as positive when the amount of antigen expression was ≥20%. Representative flow cytometry plots of bone marrow blasts from AML patients in the *DNMT3A*-mutated/CD7-positive (CD7+) group, the *DNMT3A*-mutated/CD7-negative (CD7-) group, the *DNMT3A*-wild-type/CD7+ group, and the *DNMT3A*-wild-type/CD7- group are displayed in **Supplementary Figure 1**.

### 2.3 Molecular biological testing

Molecular biological examinations were performed by next-generation sequencing (NGS). Leukemic mutation genes such as *FLT3-ITD*, *NPM1*, *KIT*, *CEBPA*, *DNMT3A*, *IDH1*, *IDH2*, *TET2*, *EZH2*, *RUNX1*, *ASXL1*, *PHF6*, *TP53*, *SF3B1*, *SRSF2*, *U2AF1*, *BCOR*, *ZRSR2*, *NRAS*, *CBL*, *SETBP1*, *ETV6* and *JAK2* were analyzed.

### 2.4 Treatment and follow-up

Patients received standard induction chemotherapy regimens based on disease and risk stratification (24, 25), with doses slightly adjusted for each patient. The induction regimens for each group of patients are summarized in **Supplementary Table 1**. Induction chemotherapy regimens included IA (idarubicin 8-12 mg/m^2^ for 3 days, cytarabine 100-200 mg/m^2^ for 7 days), DA (daunorubicin 40-60 mg/m^2^ for 3 days, cytarabine 100-200 mg/m^2^ for 7 days), MA (mitoxantrone 6-8 mg/m^2^ for 3 days, cytarabine 100-200 mg/m^2^ for 7 days), or CAG+D (decitabine 20 mg/m^2^/d, d1-5, aclarubicin 20 mg/m^2^/d, d3-6, cytarabine 25 mg/m^2^/q12h, d7-14, and G-CSF, 300 μg/d, d1-14). Patients in complete remission (CR) continued to undergo induction chemotherapy with medium- or high-dose cytarabine, while those in partial remission (PR) or nonremission (NR) received a second cycle of induction chemotherapy or salvage therapy. Based on the availability of donors, patients with intermediate and adverse prognoses underwent hematopoietic stem cell transplantation (HSCT).

### 2.5 Definitions of treatment response and survival time

CR was defined as <5% blasts in BM, no blasts with Auer bodies, no blasts in peripheral blood, no extramedullary leukemia, neutrophil count ≥1.0×10^9^/L, and PLT count ≥100× 10^9^/L. CR with incomplete hematologic recovery (CRi) was defined as meeting all CR criteria, except residual neutropenia (<1.0×10^9^/L) or thrombocytopenia (<100×10^9^/L). PR was defined as the proportion of BM blasts between 5% and 25%, which should be at least 50% lower than before therapy, and the blood cell count must be within the range of CR. NR was defined as failure to achieve CR, CRi or PR after chemotherapy. The primary study endpoints were overall survival (OS), measured as the time from diagnosis to the date of death from any cause or the date of the last follow-up, and relapse-free survival (RFS), measured as the time from the date of CR or CRi to the date of hematologic relapse, death from any cause, or the date of the last follow-up (3).

### 2.6 Statistical analysis

The comparisons between groups were made using Kruskal-Wallis rank-sum test and one-way ANOVA for continuous variables, which are presented as medians (ranges). Fisher’s exact test or the chi-square test was used to compare categorical variables. The Kaplan−Meier method was used to examine OS and RFS, and the log rank test was used to compare group differences. To examine the parameters influencing OS and RFS, univariate and multivariate Cox proportional hazards regression were performed. All statistical analyses were carried out using SPSS software version 21.0, and graphs were created using GraphPad Prism version 9.0. Two-sided p values <0.05 were considered statistically significant. Additionally, OncoPrint was utilized to identify and visualize gene mutations in AML patients.

## 3 Results

### 3.1 Clinical characteristics

This study comprised 297 patients with a median age of 48 years (range: 5-79 years), including 172 men and 125 women. In the study population, the median and range of WBC and PLT counts, Hb levels, and percentage of blasts in BM and PB were 15.88 (0.43-384.87)×10^9^/L, 38.5 (2-579)× 10^9^/L, 76 (35-147) g/L, 62.0% (12.4%-96.0%), and 43% (0-97%), respectively. According to the mutation of *DNMT3A* and the expression of CD7, the patients were divided into the following four groups: the *DNMT3A*-mutated/CD7+ group (n=21), the *DNMT3A*-mutated/CD7- group (n=44), the *DNMT3A*-wild-type/CD7+ group (n=104), and the *DNMT3A*-wild-type/CD7-group (n=128). We found that the *DNMT3A*-mutated/CD7+ group had significantly higher initial WBC counts than the other three groups (all *p*<0.05, **Table 1**). The age of the *DNMT3A*-mutated patients was significantly higher than that of the *DNMT3A*-wild-type patients (*p*=0.001). The *DNMT3A*-mutated patients had higher initial PLT counts and BM blast ratios (*p*<0.001, *p*=0.030). The rate of the FAB-M5 subtype was higher in *DNMT3A*-mutated AML patients than in *DNMT3A*-wild-type AML patients (35.4% vs. 15.1%, *p*<0.001), and the rate of the FAB-M2 subtype was lower than that in *DNMT3A*-wild-type patients (38.5% vs. 59.5%, *p*=0.003). Patients were categorized into favorable, intermediate and adverse risk groups according to the 2022 ELN stratification, with no significant differences among the four groups (*p*>0.05). Moreover, no differences in any other baseline characteristics were discovered among these four groups (*p*>0.05, **Table 1**).

**Table 1:** Clinical characteristics of patients with AML classified according to the status of *DNMT3A* mutation and CD7 expression.

Patient parameters	*DNMT3A*-mutated/CD7+(n=21)	*DNMT3A*-mutated/CD7-(n=44)	*DNMT3A*-wild-type/CD7+(n=104)	*DNMT3A*-wild-type/CD7-(n=128)	*p* value
Sex, male/female	10/11	28/16	59/45	75/53	0.662
Median age, years (range)	54 (28-73)	53 (23-74)	44 (7-76)	46.5 (5-79)	0.005
Age, number (%)					0.054
≤14 years	0 (0.0)	0 (0.0)	7 (6.7)	10 (7.8)	
15-59 years	13 (61.9)	27 (61.4)	78 (75.0)	85 (66.4)	
≥60 years	8 (38.1)	17 (38.6)	19 (18.3)	33 (25.8)	
Median WBC, ×10^9^/L (range)	47.14 (0.95-327.78)	20.7 (0.87-348.46)	19.13 (0.43-384.87)	11.45 (0.78-243.07)	0.033
Median Hb, g/L (range)	68 (37-113)	83.1 (46-115)	79 (35-147)	75 (36-131)	0.069
Median PLT, ×10^9^/L (range)	44 (8-295)	60.5 (7-248)	28 (3-196)	40 (2-579)	<0.001
Median LDH, U/L (range)	772 (175-6347)	367 (111-4241)	402 (130-3071)	430 (69-6230)	0.211
Median BM blast, % (range)	63.2 (24.4-90.0)	68.2 (14.4-93.6)	60.8 (12.4-94.0)	59.0 (15.2-96.0)	0.174
Median PB blast, % (range)	51.5 (0-88)	42 (0-93)	51 (0-97)	40 (0-95)	0.121
FAB category					
M0	2 (9.5)	2 (4.5)	3 (2.9)	1 (0.8)	0.061
M1	0 (0.0)	2 (4.5)	5 (4.8)	5 (3.9)	0.908
M2	10 (47.6)	15 (34.1)	68 (65.4)	70 (54.7)	0.005
M4	4 (19.0)	5 (11.4)	14 (13.5)	16 (12.5)	0.845
M5	3 (14.3)	20 (45.5)	7 (6.7)	28 (21.9)	<0. 001
M6	0 (0.0)	0 (0.0)	0 (0.0)	1 (0.8)	1
M7	0 (0.0)	0 (0.0)	1 (1.0)	0 (0.0)	0.569
Unclassifiable	2 (9.5)	0 (0.0)	6 (5.8)	7 (5.5)	0.271
2022-ELN risk stratification/n (%)					
Favorable	4 (19.0)	16 (36.4)	25 (24.0)	48 (37.5)	0.078
Intermediate	12 (57.1)	15 (34.1)	41 (39.4)	40 (31.3)	0.117
Adverse	5 (23.8)	13 (29.5)	38 (36.5)	40 (31.3)	0.623
HSCT/n (%)	0 (0.0)	9 (20.5)	25 (24.0)	28 (21.9)	0.100

WBC, white blood cell; Hb, hemoglobin; PLT, platelet; LDH, lactate dehydrogenase; BM, bone marrow; PB, peripheral blood; FAB, French-American-British classification; HSCT, hematopoietic stem cell transplantation.

### 3.2 Correlation between CD7 expression and other immunophenotypes

Our results showed that the positive expression proportions of CD34, CD117 and CD123 were significantly higher in the CD7+ group than in the CD7- group (*p*<0.05, **Table 2**); the positive expression proportions of CD4, CD56 and CD19 were significantly lower in the CD7+ group than in the CD7- group (*p*<0.05; **Table 2**). The differences in the proportions of CD38, HLA-DR, CD15, CD33, CD2, CD64 and CD71 expression between the two groups were compared separately, and the results were not significantly different (*p*>0.05, **Table 2**).

**Table 2:** Correlation between CD7 expression and other immunophenotypes.

immunophenotype/n (%)	CD7+ (n=125)	CD7- (n=172)	*p* value
CD34	107 (85.6)	105 (61.0)	<0.001
CD117	116 (92.8)	142 (82.6)	0.010
CD38	116 (92.8)	157 (91.3)	0.635
CD13	118 (94.4)	154 (89.5)	0.136
CD123	117 (93.6)	143 (83.1)	0.007
CD64	70 (56.0)	109 (63.4)	0.200
CD4	28 (22.4)	65 (37.8)	0.005
CD71	21 (16.8)	32 (18.6)	0.688
CD56	35 (28.0)	74 (43.0)	0.008
CD15	56 (44.8)	75 (43.6)	0.838
CD33	120 (96.0)	161 (93.6)	0.367
HLA-DR	115 (92.0)	146 (84.9)	0.064
CD19	2 (1.6)	21 (12.2)	0.001
CD2	12 (9.6)	16 (9.3)	0.931

### 3.3 Correlation of *DNMT3A* mutation with other molecular genetics

**Figure 1:**
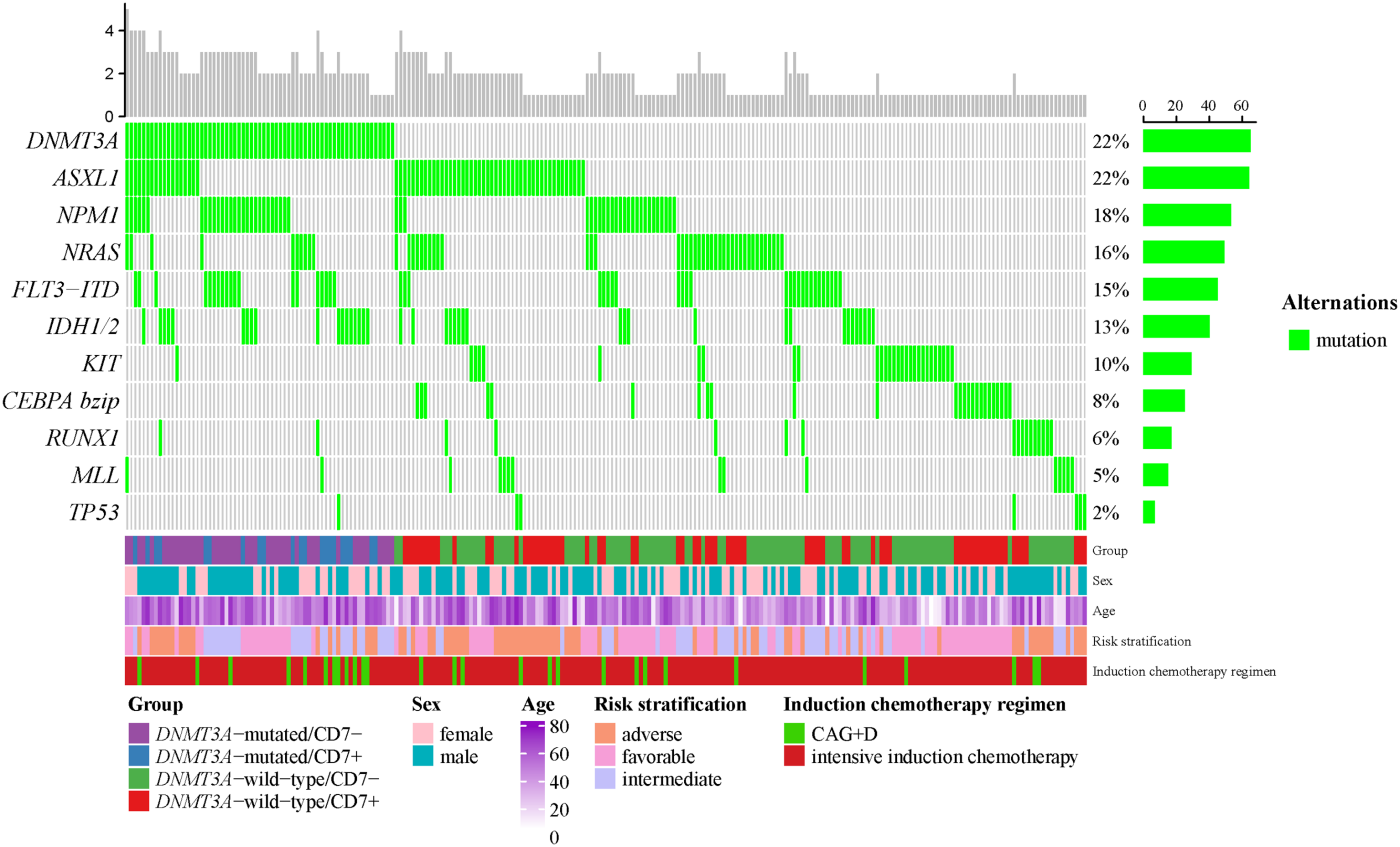
The OncoPrint of genes in AML. Mutations were colored as green. The upper barplot indicates the number of genetic mutation per patient, while the right barplot shows the proportion of genetic mutations per gene. The group, sex, age, 2022-ELN risk stratification and induction chemotherapy regimen were added as annotations for the patients.

According to the 2022 ELN genetic risk stratification, we divided AML patients into favorable (n=36), intermediate (n=242), and adverse (n=19) karyotype groups. Within the intermediate karyotype group, the *CEBPA bZIP* mutation rate was significantly lower in the *DNMT3A*-mutated group (0.0%) than in the *DNMT3A*-wild-type group (12.0%); the *FLT3-ITD* mutation rate (32.2%), *NPM1* mutation rate (47.5%) and *IDH1/2* mutation rate (28.8%) were significantly higher in the *DNMT3A*-mutated group than in the *DNMT3A*-wild-type group (11.5%, 13.1%, 10.9%), the differences were statistically significant (*p*<0.05, **Table 3**). However, no differences in gene mutation rates were observed between the *DNMT3A*-mutated group and the *DNMT3A*-wild-type group in the favorable and adverse karyotype groups (**Supplementary Table 2**). Our further analysis revealed that the *DNMT3A*-mutated/CD7+ group still exhibited a higher *FLT3-ITD* mutation rate compared with the non-*DNMT3A*mut/CD7+ group (**Supplementary Table 3**). In addition, we generated an oncoprint to display the distribution of gene mutations in AML patients (**Figure 1**).

**Table 3:** Correlation of *DNMT3A* mutation status with other molecular genetic mutations in intermediate karyotype.

Gene mutation/n (%)	*DNMT3A*-mutated (n=59)	*DNMT3A*-wild-type (n=183)	*p* value
*RUNX1*	2 (3.4)	15 (8.2)	0.335
*FLT3-ITD*	19 (32.2)	21 (11.5)	<0.001
*ASXL1*	15 (25.4)	37 (20.2)	0.397
*CEBPA bZIP*	0 (0.0)	22 (12.0)	0.005
*NPM1*	28 (47.5)	24 (13.1)	<0.001
*TP53*	1 (1.7)	4 (2.2)	1.000
*MLL*	2 (3.4)	12 (6.6)	0.558
*KIT*	0 (0.0)	6 (3.3)	0.354
*NRAS*	9 (15.3)	31 (16.9)	0.762
*IDH1/2*	17 (28.8)	20 (10.9)	0.001

### 3.4 Prognostic impact of *DNMT3A* mutation and CD7 expression on AML patients respectively

**Figure 2:**
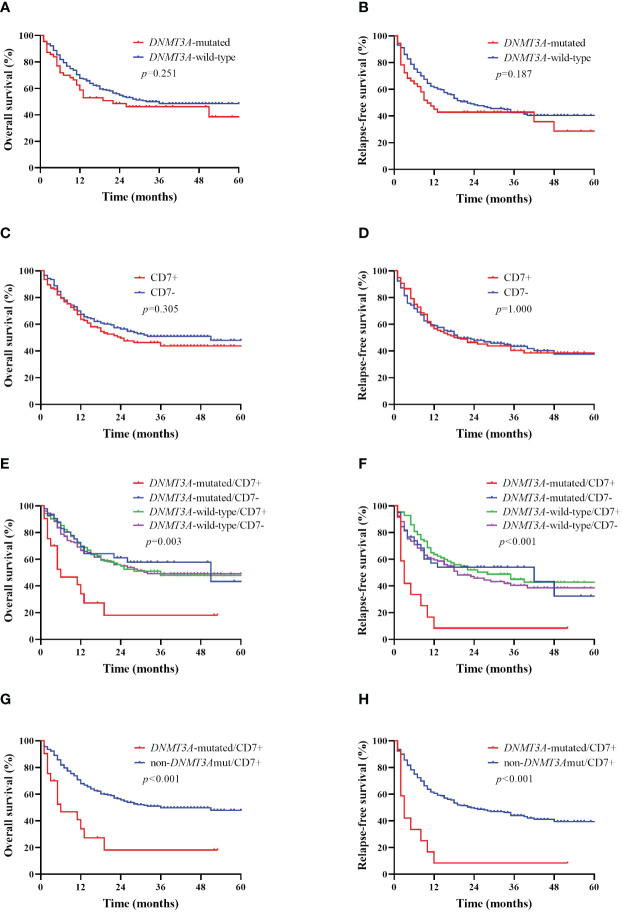
Kaplan–Meier curves of overall survival (OS) and relapse-free survival (RFS) in AML patients according to *DNMT3A* mutation and CD7 expression. **(A, B)** Kaplan–Meier curves of OS and RFS in *DNMT3A* mutation and *DNMT3A* wild-type AML patients. **(C, D)** Kaplan–Meier curves of OS and RFS in CD7+ and CD7- AML patients. **(E, F)** Kaplan–Meier curves of OS and RFS of AML patients in the *DNMT3A*-mutated/CD7+ group, *DNMT3A*-mutated/CD7- group, *DNMT3A*-wild-type/CD7+ group and the *DNMT3A*-wild-type/CD7- group. **(G, H)** Kaplan–Meier curves of OS and RFS in *DNMT3A*-mutated/CD7+ and non-*DNMT3A*mut/CD7+ AML patients.

AML patients were divided into two groups based on their *DNMT3A* mutation status; the median OS time was 22 months for *DNMT3A*-mutated patients and 32 months for *DNMT3A*-wild-type patients, with no statistically significant difference (*p*>0.05, **Figure 2A**); similarly, the median RFS of the *DNMT3A*-mutated group was not significantly different from that of the *DNMT3A*-wild-type group (10 months vs. 22 months, *p*>0.05, **Figure 2B**). AML patients were divided into two groups according to CD7 expression; the median OS time was 24 months in the CD7+ group and 51 months in the CD7- group, and the difference in OS between the two groups was not significantly different (*p*>0.05, **Figure 2C**); the median RFS time was 20 months and 19 months in the CD7+ and CD7- groups, respectively, and the difference was also not significantly different (*p*>0.05, **Figure 2D**).

### 3.5 Prognostic impact of CD7+ with *DNMT3A* mutation in AML patients

The median OS time for patients in the *DNMT3A*-mutated/CD7+ group was 6 months, whereas that in the *DNMT3A*-mutated/CD7- group was 51 months (*p*=0.003, **Figure 2E**); similarly, the median OS in the *DNMT3A*-mutated/CD7+ group was significantly shorter than that in the *DNMT3A*-wild-type/CD7+ and *DNMT3A*-wild-type/CD7- groups (6 months vs. 36 months, *p*=0.001; 6 months vs. 32 months, *p*=0.001, **Figure 2E**). In addition, the median RFS was significantly shorter in the *DNMT3A*-mutated/CD7+ group than in the other three groups (3 months vs. 42 months, *p*=0.003; 3 months vs. 28 months, *p*<0.001; 3 months vs. 19 months, *p*<0.001, **Figure 2F**). Therefore, we reclassified AML patients into the *DNMT3A*-mutated/CD7+ group (n=21) and the other (non-*DNMT3A*mut/CD7+) group (n=276) based on whether they had concomitant *DNMT3A* mutation and positive CD7 expression. Comparing the OS and RFS of patients in the two groups separately, we found that the *DNMT3A*-mutated/CD7+ group had significantly shorter OS and RFS (*p*<0.001, **Figures 2G, H**).

### 3.6 Effect of CD7+ with *DNMT3A* mutation on the initial treatment response in AML patients

Among the entire cohort of 297 patients, CR (CR/CRi) was achieved in 233 patients (78.5%) after two cycles of induction chemotherapy.

CR was achieved in 12 patients (57.1%) in the *DNMT3A*-mutated/CD7+ group compared to 221 patients (80.1%) in the non-*DNMT3A*mut/CD7+ group, and the CR rate was significantly lower in the *DNMT3A*-mutated/CD7+ group than in the non-*DNMT3A*mut/CD7+ group (*p*=0.003). Of the 233 patients with CR, 97 experienced disease recurrence, including 9 (75.0%) in the *DNMT3A*-mutated/CD7+ group and 88 (39.8%) in the non-*DNMT3A*mut/CD7+ group. Patients in the *DNMT3A*-mutated/CD7+ group presented a higher risk of recurrence (*p*=0.032, **Table 4**). Consistent with this, we found that *DNMT3A*-mutated/CD7+ patients still showed a low CR rate and a high relapse rate among patients who received intensive induction therapy (**Supplementary Table 4**).

**Table 4:** Treatment response to the first and second cycles of induction chemotherapy between the two groups.

Response/n (%)	*DNMT3A*-mutated /CD7+(n=21)	Non-*DNMT3A*mut/CD7+(n=276)	*p* value
First cycle			0.306
CR/CRi	10 (47.6)	172 (62.3)	
PR	6 (28.6)	49 (17.8)	
NR	5 (23.8)	55 (19.9)	
Second cycle			0.003
CR/CRi	12 (57.1)	221 (80.1)	
PR	6 (28.6)	17 (6.2)	
NR	3 (14.3)	38 (13.8)	
Relapse	9 (75.0)	88 (39.8)	0.032

CR, complete remission; CRi, CR with incomplete hematologic recovery; PR, partial remission; NR, no remission.

### 3.7 Univariate and multivariable analyses of clinical prognostic factors

We analyzed various prognostic factors affecting OS and RFS in AML patients. Univariate Cox regression analysis showed that age >60 years, WBC count ≥100 × 10^9^/L, no HSCT, and *DNMT3A*-mutated/CD7+ were risk factors for poorer OS and RFS in patients with newly diagnosed AML. Multifactorial Cox regression analysis showed that *DNMT3A*-mutated/CD7+ remained significantly associated with shorter OS and RFS (**Table 5**). In addition, we further analyzed the factors affecting prognosis in patients who did not undergo HSCT and those who received intensive induction therapy, and multifactorial Cox regression analysis still suggested that *DNMT3A*-mutated/CD7+ was an independent risk factor for AML patients (**Supplementary Table 5**).

**Table 5:** Univariate and multivariate analysis of OS and RFS in AML patients.

Variables	Univariate				Multivariate			
	OS		RFS		OS		RFS	
	HR (95% CI)	*p* value	HR (95% CI)	*p* value	HR (95% CI)	*p* value	HR (95% CI)	*p* value
Age (>60years)	1.812 (1.264-2.597)	0.001	1.568 (1.065-2.309)	0.023	1.532 (1.065-2.205)	0.022	1.361 (0.921-2.011)	0.122
WBC (≥100×10^9^/L)	1.928 (1.239-2.999)	0.004	1.612 (1.002-2.594)	0.049	2.219 (1.404-3.507)	0.001	1.779 (1.094-2.893)	0.020
HB (<100 g/L)	1.585 (0.941-2.668)	0.083	1.299 (0.801-2.109)	0.289				
PLT (<20×10^9^/L)	0.992 (0.624-1.362)	0.683	0.920 (0.625-1.353)	0.671				
LDH (≥700U/L)	1.361 (0.952-1.946)	0.091	1.078 (0.740-1.570)	0.695				
Adverse risk	1.385 (0.978-1.961)	0.067	1.230 (0.857-1.767)	0.262				
HSCT	0.239 (0.132-0.432)	<0.001	0.372 (0.231-0.598)	<0.001	0.250 (0.136-0.457)	<0.001	0.388 (0.239-0.630)	<0.001
CD7	1.187 (0.851-1.657)	0.312	1.000 (0.712-1.404)	1.000				
*DNMT3A*	1.259 (0.844-1.878)	0.258	1.305 (0.871-1.955)	0.197				
*DNMT3A*-mutated/CD7+	2.707 (1.551-4.724)	<0.001	3.329 (1.786-6.206)	<0.001	1.792 (1.013-3.170)	0.045	2.488 (1.320-4.689)	0.005

HR, hazard ratio; CI, confidence interval; OS, overall survival; RFS, relapse-free survival; HSCT, hematopoietic stem cell transplantation.

## 4 Discussion

In this study, the importance of *DNMT3A* mutation combined with CD7 expression for the precise stratification of AML patients was evaluated. Our results showed that CD7+ AML patients with *DNMT3A* mutations had lower CR rates and higher relapse rates. More importantly, our data showed for the first time that OS and RFS were significantly shorter in AML patients with *DNMT3A* mutations combined with CD7 expression, whereas *DNMT3A* mutations or CD7-positive expression alone had no significant effect on prognosis.

The CD7 antigen is a T-cell-related antigen that is expressed on thymocytes, T cells, and natural killer cells as well as lymphocytes and myeloid progenitors in healthy individuals (18), whereas 30% of AML patients exhibit positive CD7 expression (16–18). There is still controversy about whether CD7+ indicates poor prognosis of AML. Our data showed that there was no significant difference in OS and RFS between CD7+ AML patients and CD7- AML patients, which was similar to that reported by Fang et al. (23), suggesting that CD7 expression may not be related to the prognosis of AML. However, Ogata et al. found differences in the prognostic impact of CD7+ in AML patients under different cytogenetic conditions (18). Therefore, the prognostic significance of CD7 positivity may need further subgroup analysis.

Some studies have shown that *DNMT3A* gene mutations precede the AML stage (26, 27). Therefore, the impact of *DNMT3A* gene mutations on the prognosis of AML patients should be given sufficient attention. In our analysis, there was no significant difference in OS and RFS between the *DNMT3A*-mutated group and the *DNMT3A*-wild-type group. Univariate and multivariate analysis showed that *DNMT3A* mutation was also unrelated with OS and EFS. The present study is consistent with previous studies showing that *DNMT3A* mutation cannot be used as an independent prognostic marker for AML patients (28, 29). However, some studies have found that *DNMT3A* mutations in AML patients affect patient prognosis and are negatively associated with patient prognosis (6, 7), and this adverse prognostic effect may be limited to specific types of AML groups (8–11). Therefore, we further analyzed their prognostic impact in combination with specific immunophenotypes.

Interestingly, when we combined *DNMT3A* mutation with CD7-positive expression, we found that patients in the *DNMT3A*-mutated/CD7+ group had significantly shorter OS and RFS compared with patients in the other three groups. However, no significant differences were observed in OS and RFS among the remaining three groups. Therefore, we reclassified AML patients into *DNMT3A*-mutated/CD7+ and non-*DNMT3A*mut/CD7+ groups. Notably, we found that patients in the *DNMT3A*-mutated/CD7+ group had lower CR rates, higher relapse rates, and shorter OS and RFS. Multivariate regression analysis also showed that CD7+ with *DNMT3A* mutation was a Powerful Predictor for poor prognosis in AML patients. This result has not yet been reported in the literature.

The reason for the poorer prognosis of *DNMT3A*-mutated/CD7+ patients is currently unknown. We hypothesize that it is related to the following factors. First, we observed higher WBC counts at the initial diagnosis in patients with *DNMT3A*-mutated/CD7+, potentially explaining their poorer prognosis. In addition, our data showed that AML patients with *DNMT3A* mutations frequently carry *NPM1* and *FLT3-ITD* mutations, which is consistent with previous studies (9, 11, 29, 30). Studies have shown that AML patients with mutations in all three *DNMT3A*/*FLT3*/*NPM1* genes have a higher disease load and lower cumulative overall survival (31–33). Our data also showed that the *DNMT3A* mutation group was associated with lower *CEBPA bZIP* mutation rates. Increasing evidence suggests that *CEBPA bZIP* in-frame mutations are associated with favorable AML prognoses and have been incorporated into ELN risk stratification (3, 34–36). Therefore, we infer this is related to the poor prognosis of *DNMT3A*-mutated/CD7+ patients. Furthermore, we also found that the positive expression proportion of CD34, CD117, and CD123 was significantly higher in the CD7+ AML group than in the CD7- AML group, and the proportion of positive expression of CD4- was lower than in the CD7- AML group. CD34 and CD117 are precursor markers, and previous studies have shown that CD34-positive expression is associated with lower CR rates (17, 37–39). CD123 is a specific antigen on the surface of leukemia stem cells (LSCs) (40), and several studies have found that CD123 is an independent risk factor for CR and OS in AML patients (40, 41). Relevant literature shows that AML leukemia cells with CD4 expression originate from a relatively mature stage (42). Our study showed that CD7+ was associated with high expression of immature phenotype and low expression of mature phenotype. This association may contribute to the poorer clinical outcomes of AML patients with *DNMT3A*-mutated/CD7+. However, the underlying mechanism of this association requires further exploratory studies.

As this study was a retrospective study, there may be a small number of cases in some groups after grouping, which may have biased the data. In addition, the heterogeneity of treatment regimens may confound the results of this study. The data we collected showed no patients in the *DNMT3A-*mutated/CD7+ group who underwent HSCT. We consider that the reason for this phenomenon may be related to the small sample size in this subgroup analysis. Furthermore, not undergoing HSCT may also be influenced by the patient’s financial status, HLA matching, and complications. Therefore, large-scale, multicenter prospective studies are needed to further validate the prognostic value of CD7+ with *DNMT3A* mutation.

## 5 Conclusions

In conclusion, we found that CD7+ with *DNMT3A* mutation is an effective prognostic marker in AML patients and has a negative impact on the OS and RFS of these patients. Our results reveal the clinical value of immunophenotyping for the precise molecular genetic stratification of AML.

## Data availability statement

The raw data supporting the conclusions of this article will be made available by the authors, without undue reservation.

## Ethics statement

The studies involving humans were approved by the Medical Ethical Committee of Henan Provincial People’s Hospital (2021-140-02). The studies were conducted in accordance with the local legislation and institutional requirements. Written informed consent for participation was not required from the participants or the participants’ legal guardians/next of kin in accordance with the national legislation and institutional requirements.

## Author contributions

YB: Conceptualization, Writing – original draft. XS: Investigation, Formal analysis, Writing – original draft. ML: Investigation, Writing – review & editing. XN: Resources, Writing – review & editing. WC: Resources, Writing – review & editing. JN: Writing – review & editing. XX: Writing – review & editing. YC: Writing – review & editing. KS: Supervision, Writing – review & editing.
