# Supplementary material for: CD7-positive leukemic blasts with DNMT3A mutations predict poor prognosis in patients with acute myeloid leukemia
Source: Front Oncol. 2024 Mar 21;14:1342998. doi: 10.3389/fonc.2024.1342998 (PMC10991683; doi:10.3389/fonc.2024.1342998)
Supplement: Supplementary file 1 [file DataSheet_1.docx]

Supplementary Material

# Supplementary Figure and Tables

## Supplementary Figure


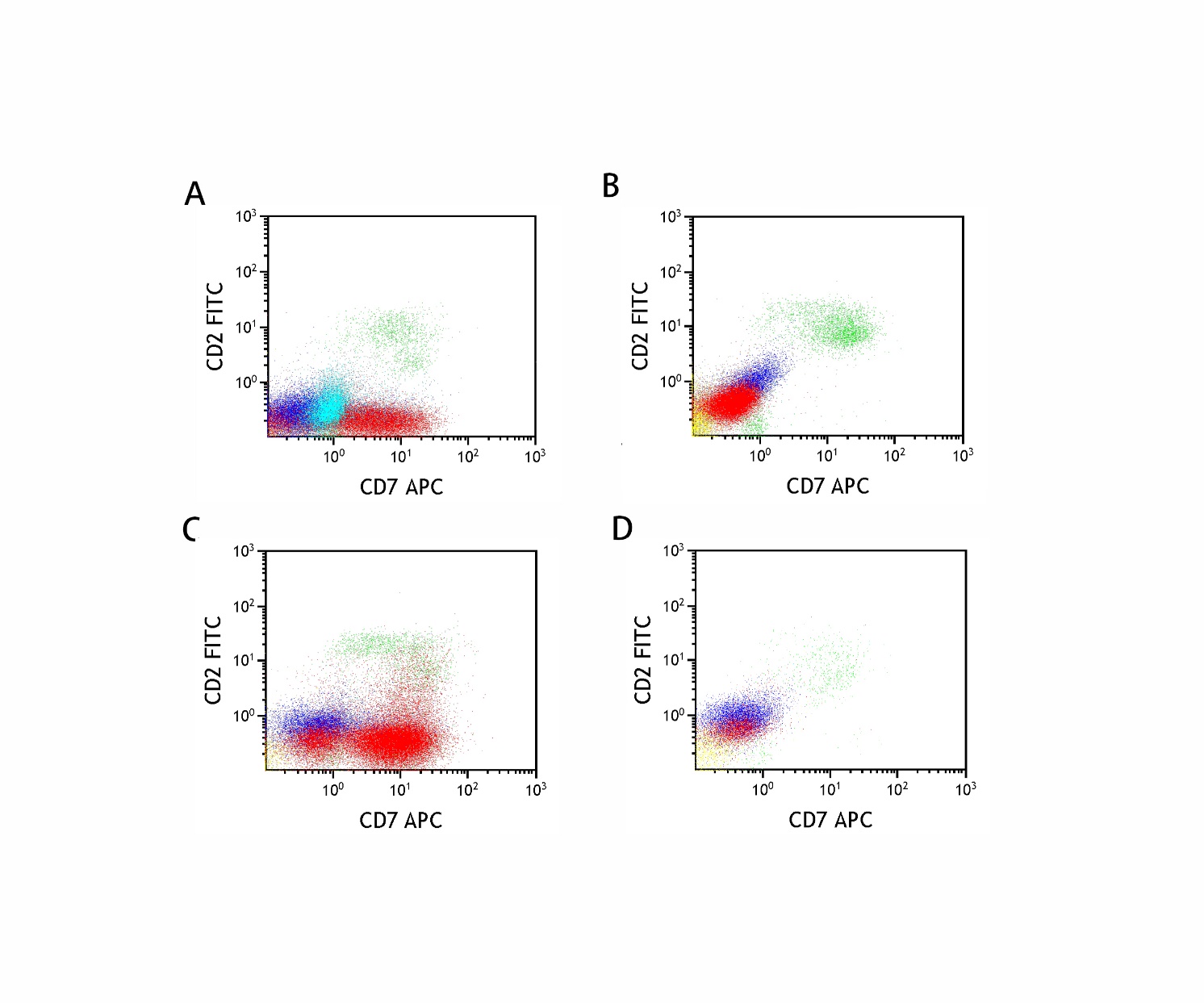


**Supplementary FIGURE 1 |** Representative flow cytometry plots of bone marrow blasts from AML patients in the *DNMT3A*-mutated/CD7+ (A), the *DNMT3A*-mutated/CD7- (B), the *DNMT3A*-wild-type/CD7+ group (C), and the *DNMT3A*-wild-type/CD7- (D) groups. Red represents bone marrow blasts.

## Supplementary Tables

**SUPPLEMENTARY TABLE 1** | Induction chemotherapy regimens of patients with AML classified according to the status of *DNMT3A* mutation and CD7 expression.

| Induction chemotherapy regimen/n (%) | Total（n=297） | *DNMT3A*-mutated/CD7+  (n=21) | *DNMT3A*-mutated/CD7-  (n=44) | *DNMT3A*-wild-type/  CD7+  (n=104) | *DNMT3A*-wild-type  /CD7-  (n=128) | *p* value |
| --- | --- | --- | --- | --- | --- | --- |
| CAG+D | 43(14.5) | 4(19.0) | 8(18.2) | 15(14.4) | 16(12.5) | 0.742 |
| Intensive induction chemotherapy | 254(85.5) | 17(81.0) | 36(81.8) | 89(85.6) | 112(87.5) |  |

**SUPPLEMENTARY TABLE 2** | Correlation of *DNMT3A* mutation status with other molecular genetic mutations.

| Gene mutation/n (%) | Favorable karyotype | | | Intermediate karyotype | | | Adverse karyotype | | |
| --- | --- | --- | --- | --- | --- | --- | --- | --- | --- |
|  | *DNMT3A*-mutated | *DNMT3A*-wild-type | *p* value | *DNMT3A*-mutated | *DNMT3A*-wild-type | *p* value | *DNMT3A*-mutated | *DNMT3A*-wild-type | *p* value |
|  | (n=3) | (n=33) |  | (n=59) | (n=183) |  | (n=3) | (n=16) |  |
| *RUNX1* | 0(0.0) | 0(0.0) |  | 2(3.4) | 15(8.2) | 0.335 | 0(0.0) | 0(0.0) |  |
| *FLT3-ITD* | 0(0.0) | 3(9.1) | 1.000 | 19(32.2) | 21(11.5) | <0.001 | 0(0.0) | 2(12.5) | 1.000 |
| *ASXL1* | 2(66.7) | 5(15.2) | 0.090 | 15(25.4) | 37(20.2) | 0.397 | 1(33.3) | 4(25.0) | 1.000 |
| *CEBPA* *bZIP* | 0(0.0) | 3(9.1) | 1.000 | 0(0.0) | 22(12.0) | 0.005 | 0(0.0) | 0(0.0) |  |
| *NPM1* | 0(0.0) | 0(0.0) |  | 28(47.5) | 24(13.1) | <0.001 | 0(0.0) | 1(6.3) | 1.000 |
| *TP53* | 0(0.0) | 0(0.0) |  | 1(1.7) | 4(2.2) | 1.000 | 0(0.0) | 2(12.5) | 1.000 |
| *MLL* | 0(0.0) | 0(0.0) |  | 2(3.4) | 12(6.6) | 0.558 | 0(0.0) | 1(6.3) | 1.000 |
| *KIT* | 1(33.3) | 20(60.6) | 0.559 | 0(0.0) | 6(3.3) | 0.354 | 0(0.0) | 2(12.5) | 1.000 |
| *NRAS* | 1(33.3) | 3(9.1) | 0.305 | 9(15.3) | 31(16.9) | 0.762 | 0(0.0) | 5(31.3) | 0.530 |
| *IDH1/2* | 0(0.0) | 1(3.0) | 1.000 | 17(28.8) | 20(10.9) | 0.001 | 1(33.3) | 1(6.3) | 0.298 |

**SUPPLEMENTARY TABLE 3 |** Differences in gene mutations between the DNMT3A-mutated/CD7+ group and the non-DNMT3Amut/CD7+ group in intermediate karyotype.

| Gene mutation/n (%) | *DNMT3A*-mutated/CD7+  (n=20) | Non-*DNMT3A*mut/CD7+  (n=222) | *p* value |
| --- | --- | --- | --- |
| *RUNX1* | 1(5.0) | 16(7.2) | 1.000 |
| *FLT3-ITD* | 9(45.0) | 31(14.0) | 0.001 |
| *ASXL1* | 4(20.0) | 48(21.6) | 1.000 |
| *CEBPA bZIP* | 0(0.0) | 22(9.9) | 0.284 |
| *NPM1* | 7(35.0) | 45(20.3) | 0.211 |
| *TP53* | 0(0.0) | 5(2.3) | 1.000 |
| *MLL* | 1(5.0) | 13(5.9) | 1.000 |
| *KIT* | 0(0.0) | 6(2.7) | 1.000 |
| *NRAS* | 3(15.0) | 37(16.7) | 1.000 |
| *IDH1/2* | 5(25.0) | 32(14.4) | 0.350 |

**SUPPLEMENTARY TABLE 4** | Treatment response to the first and second cycles of intensive induction chemotherapy between the two groups.

| Response/n (%) | *DNMT3A*-mutated/CD7+  (n=21) | Non-*DNMT3A*mut/CD7+  (n=276) | *p* value |
| --- | --- | --- | --- |
| First cycle |  |  | 0.216 |
| CR/CRi | 8(47.1) | 158(66.7) |  |
| PR | 4(23.5) | 36(15.2) |  |
| NR | 5(29.4) | 43(18.1) |  |
| Second cycle |  |  | 0.013 |
| CR/CRi | 10(58.8) | 195(82.3) |  |
| PR | 4(23.5) | 12(5.1) |  |
| NR | 3(17.6) | 30(12.7) |  |
| Relapse | 8(80.0) | 77(38.9) | 0.024 |

CR: complete remission; CRi: CR with incomplete hematologic recovery; PR: partial remission; NR: no remission.

**SUPPLEMENTARY TABLE 5 |** Univariate and multivariate analysis of OS and RFS in AML patients, except those who received HSCT.

| Variables | Univariate | | | | Multivariate | | | |
| --- | --- | --- | --- | --- | --- | --- | --- | --- |
|  | OS | | RFS | | OS | | RFS | |
|  | HR  (95% CI) | *p* value | HR  (95% CI) | *p* value | HR  (95% CI) | *p* value | HR  (95% CI) | *p* value |
| Age (>60 years) | 1.792  (1.249-2.571) | 0.002 | 1.548  (1.050-2.281) | 0.027 | 1.791  (1.246-2.576) | 0.002 | 1.526  (1.034-2.251) | 0.033 |
| WBC (≥100×10^9^/L) | 2.020  (1.297-3.146) | 0.002 | 1.704  (1.059-2.744) | 0.028 | 1.956  (1.247-3.067) | 0.003 | 1.624  (1.004-2.626) | 0.048 |
| HB  (<100 g/L) | 1.551  (0.907-2.652) | 0.109 | 1.253  (0.763-2.057) | 0.373 |  |  |  |  |
| PLT (<20×10^9^/L) | 0.924  (0.622-1.372) | 0.694 | 0.919  (0.621-1.359) | 0.673 |  |  |  |  |
| LDH (≥700U/L) | 1.379  (0.963-1.973) | 0.079 | 1.094  (0.751-1.595) | 0.639 |  |  |  |  |
| Adverse risk | 1.420  (0.998-2.020) | 0.052 | 1.259  (0.871-1.819) | 0.221 |  |  |  |  |
| CD7 | 1.199  (0.857-1.678) | 0.289 | 1.007  (0.715-1.420) | 0.967 |  |  |  |  |
| *DNMT3A* | 1.286  (0.861-1.919) | 0.219 | 1.337  (0.891-2.005) | 0.160 |  |  |  |  |
| *DNMT3A*-mutated/CD7+ | 2.669  (1.529-4.660) | 0.001 | 3.268  (1.753-6.094) | <0.001 | 2.242  (1.272-3.951) | 0.005 | 2.989  (1.592-5.611) | 0.001 |

HR: hazard ratio; CI: confidence interval; OS: overall survival; RFS: relapse-free survival.
